# miR-301, Pleiotropic MicroRNA in Regulation of Inflammatory Bowel Disease and Colitis-Associated Cancer

**DOI:** 10.3389/fimmu.2018.00522

**Published:** 2018-03-15

**Authors:** Somesh Baranwal, Shiv Govind Rawat, Pooja Gupta

**Affiliations:** ^1^Department of Biochemistry and Microbial Science, School of Basic and Applied Science, Central University of Punjab, Bathinda, India; ^2^College of Agriculture, Guru Kashi University, Talwandi Sabo, India

**Keywords:** intestinal epithelial cells, apical junctional complex, knockout mouse model, pro-inflammatory cytokine, barrier function

Inflammatory bowel disease (IBD) is a chronic idiopathic, relapsing, and remitting immune disorders of gastrointestinal tract which consist of two forms, i.e., Crohn’s disease (affect the whole gastrointestinal tract) and ulcerative colitis (restrict to colon only). Recent data demonstrate that IBD arises from the complex interplay of genetic and environmental factor caused by a change in the intestinal microbiota (1). Innate immunity in gastrointestinal system includes epithelial cell layers that express tight cell–cell contact, secreted mucous layer which prevent entry of microbes, protective plasma protein, and circulating leukocytes, such as phagocytic macrophages, neutrophils, dendritic cells (DC), natural killer (NK) cells, NK T-cell, and innate lymphoid cells. The innate immune response also includes complement proteins, defensins, and chemokines which attract inflammatory leukocytes, lipid mediator of inflammation, bioactive amines, and enzymes to enhance inflammation (2). Following exposure to pathogens, the innate immune cells of gastrointestinal mucosa release pro-inflammatory cytokines and activate an adaptive immune response to aggravating inflammation in the intestine (3). Single layered intestinal epithelial cells (IECs) establish a semipermeable intestinal barrier (IB) which allows the dual role of transporting nutrients and avoiding the passage of pathogens. Selective permeability of IECs is achieved by apical junctional complex (AJC) proteins which comprise tight junctions, adherens junctions, and desmosomes to maintain the apicobasal cell polarity and intestinal tissue integrity (4). Dysregulation in intestinal epithelial monolayer cause abnormal interaction with immune cells and perturb the intestinal immune homeostasis which is associated with the clinical symptoms of IBD. Studies in the past decade have demonstrated the essential roles of several AJC protein, including underlying actinomyosin components, actin isoforms, and intracellular signal transducer and activator of transcription (STAT) transcription factors, and nuclear factor-κB (NF-κB) in the maintenance and remission of IBD progression (5).

Non-coding RNA (ncRNA) such as microRNA (miRNA) and long non-coding RNA (lncRNA) regulates the translation and/or stability of multiple mRNA targets either directly or by modulation of signal transduction pathways. Recent studies showed the existence of both positive and negative regulator of ncRNAs in the proliferation, migration, and survival of IEC, for example, negatively regulated ncRNA, such as miR-29b, miR-222, miR-146a, miR-195, miR-122a, miR-675, miR-429, and lncRNA H19 prevent translation of mRNA which enhances regeneration and disrupts the integrity of intestinal epithelial cell, while positively regulated ncRNA, such as miR-503 and lncRNA SPRY4-IT1 increases mucosal growth and protect the intestinal epithelial integrity (6). Further, a number of miRNAs have been implicated in the initiation, development, and progression of Crohn’s disease, ulcerative colitis, and colitis-associated cancer (CAC) with the potential to be used as a biomarker and therapeutic targets, for example, aberrant expression of miR-19b, miR-122, miR-141, and miR-210 is specifically associated with Crohn’s disease, while expression of miR-146b, miR-210, miR-223, and let-7e is dysregulated in patients with both Crohn’s disease and ulcerative colitis (7).

miR-301a is initially identified as a novel ncRNA to be upregulated in T-helper cells in response to myelin oligodendrocyte protein antigen in the multiple sclerosis (8). Further study show glycine as a novel upstream regulator of miR-301a and play a neuroprotective role through direct interaction with 3′UTR of PTEN to control its expression in cultured rat cortical neurons. Panguluri et al. showed significant upregulation of miR-301a in the heart of diabetic ventricles to regulate expression of voltage-gated potassium channel (Kv4.2) by direct interaction with 3′UTR in the diabetes mouse (db/db) model (9). Studies using human adipose-derived stem cells show the important role of miRNA-301a in the suppression of apoptosis signal-regulating kinase 1 expression. This study has provided promising result in treatment of damaged heart using mesenchymal stem cells based therapy (10). Further, miR-301a-3p is highly expressed in peripheral blood mononuclear cells (PBMCs) from rheumatoid arthritis (RA) patients and attenuate expression of protein inhibitor of activated STAT3 (PIAS3) to regulate differentiation of Th17 cells (11). Numerous reports have uncovered the critical role of miRNA-301a in proliferation, drug resistance, and metastasis of pancreatic, colon, and gastric cancer. In Ewing’s sarcoma (ES) cells, miRNA-301a regulates PTEN expression and cell proliferation in ES cell. Additionally, in breast cancer, miR-301a is upregulated in primary tumor sample with distant metastasis and maintains constitutively activated Wnt/β-catenin signaling by directly targeting PTEN (12). Further, miRNA-301a controls osteosarcoma and non-small cell lung cancer progression by targeting CDC14a and DLC1, respectively (13, 14). In osteosarcoma cells, miR-301a modulates doxorubicin resistance and targets AMP-activated protein kinase alpha 1. miR-301a expression is identified as an independent prognostic factor for survival in pancreatic cancer patients. Mechanistically, miR-301a inhibits SMAD4 expression to regulate invasion, migration *in vitro* as well as tumor growth *in vivo* in pancreatic cancer (15). Moreover, miR-301a is upregulated in hepatocellular cancer and inhibits NF-κB expression by negative regulation of homeobox gene Gax (16). Hence, miR-301a is associated with several autoimmune diseases and its expression is dysregulated in many cancers (Table 1).

**Table 1:** Major molecular targets of miR-301a and their biological functions.

Target gene	Model	Biological functions	Reference
Gax	Hepatocellular carcinoma	Modulates NF-κB expression and overexpression, promotes proliferation, migration, and inhibits apoptosis *in vivo*	(16)

DLC1	Non-small cell lung cancer	Inversely suppressed proliferation, migration, and invasion	(14)

SMAD4	Pancreatic ductal adenocarcinoma	Independent prognostic marker for lymph node metastasis. Regulates *in vitro* migration and *in vivo* tumorigenicity	(15)

PTEN and Wnt/β-catenin pathway	Breast cancer cell	Sustain constitutively activated Wnt/β-catenin activity to regulate *in vitro* and *in vivo* migration and metastasis	(12)

NKRF and PIAS3	Lung and colon cancer	Genetic ablation protects animals from DSS-induced colonic inflammation and colitis-associated cancer	(17)

CDC14a	Osteosarcoma	Increase cell cycle progression, proliferation, and migration	(13)

SNIP1	Human PBMC and inflamed mucosa from intestinal epithelial cells from inflammatory bowel disease (IBD)	Contributes to Th17 cell differentiation, alleviates TNBS-induced colitis in mice through TNFα production	(11)

BTG1	IBD and colon cancer cells	Reduces epithelial cell integrity and enhances inflammation in mouse colon and promotes tumorigenesis. Knockout mice are resistant to DSS-induced colitis and enhance intestinal barrier function	(18)

PIAS3	Autoimmune encephalomyelitis	Regulates the function of myelin-reactive T-helper type 17 cells	(8)

*miR-301a, micro-ribonucleic acid-301a; TGFBR2 gene, transforming growth factor beta receptor 2; PTEN, phosphatase and tensin homologue; NF-κB, nuclear factor κB; Gax, growth arrest-specific homeobox; DLC1, deleted in liver cancer 1; SMAD4, SMAD family member 4; STAT3, signal transducer and activator of transcription 3; CDC14a, cell division cycle 14a; AMPKα1, AMP-activated protein kinase alpha 1; PBMC, peripheral blood mononuclear cells; SNIP1, Smad nuclear interacting protein 1; BTG1, B cell translocation gene 1; TNBS 1; 2,4,6-trinitrobenzene sulfonic acid; TNFα, tumor necrosis factor alpha; PIAS3, protein inhibitor of activated STAT 3; NKRF, NF-kappa-B-repressing factor; DSS, dextran sodium sulfate; AOM-DSS, azoxymethane-dextran sodium sulfate*.

Emerging report reveal the prominent function of miR-301a in ulcerative colitis and CAC. qPCR analysis showed the upregulation of miR-301a in the inflamed mucosa and PBMCs from the matched patient with IBD compared with a healthy individual. Moreover, tumor necrosis factor-α (TNF-α) treatment increases miR-301a expression in CD4+ T-cells of IBD patients. Overexpression of pre-miR-301a in patient-derived CD4+ T-cell promotes Th17 cell differentiation and TNF-α production. Their result shows Smad nuclear-interacting protein 1 as functional downstream targets of miR-301a and diminishes trinitrobenzene sulfonic acid (TNBS)-induced colitis to decrease expression of IL-17A and TNF-α in the inflamed colon (11). Few studies have conducted on the knockout animal model to understand the molecular mechanism of miRNA in the pathobiology of inflammation-associated cancer. Ma and colleagues showed that miR-301a knockout mice inhibit KrasLA2-driven lung tumorigenesis and significantly decreased the incidence of thymic lymphomas and skin papillomae. Furthermore, miR-301a knockout mice show significantly less inflammation in DSS-induced colitis and AOM-DSS-induced colorectal cancer in comparison to their wild type. Moreover, deletion of miR-301a in bone marrow (BM)-derived cells inhibits *in vivo* tumor growth in colon cancer and miR-301a knockout impedes tumorigenesis of lewis lung carcinoma cell in a syngeneic murine xenograft model. Importantly, miR-301a modulates expression of IL-6, TNF-α, and IL-17 in LLC-1 xenograft tumors. In summary, miR-301a activates expression of both NF-κB and STAT3 in the tumor-associated immune cell to enhance pro-inflammatory condition and derived lung and colon cancer tumorigenesis (17).

Using IECs, He et al. showed that miR-301a is overexpressed in IEC from CD and UC patients. Further, IL1b stimulates miR-301a expression in colon cancer cell. Further, there is a positive correlation between miR-301a and IL1b expression in the serum of IBD patients. Luciferase promoter analysis shows that IL1b induces c-Jun phosphorylation to upregulate miR-301a expression through direct interaction of its promoter region. Lentivirus-mediated overexpression of miR-301a promotes NF-κB signaling and enhances expression of IL1b, IL6, IL8, and TNFα in IEC. Moreover, their data reveal an inverse relationship between miR-301a expression and BTG anti-proliferation factor 1 (BTG1) to regulate intestinal inflammation and CAC. To confirm their *in vitro* results, they generated miR-301a knockout mice employing CRISPR/Cas9 system and studied the role of miR-301a in protecting colitis in BTG1 transgenic mice. In DSS mouse model, miR-301a null mice develop mild colitis and show significantly less number of neutrophils, DC, and CD4 positive T-cells in comparison to BL6 wild type. Importantly, ADP ribosylation factor guanine nucleotide exchange factor 1 (BIG1) expression was decreased in IEC from DSS colitis-induced wild type compared to untreated control and BIG1 transgenic mice which are resistant to DSS-induced colitis. Moreover, in AOM/DSS-induced CAC model, miR-301a knockout show smaller and significantly less number of colonic tumor in comparison to wild type counterpart. Finally, they elucidated the protective roles of miR-301a in intestinal permeability and E-cadherin expression *in vivo* employing BM-derived chimeric mice. Wild type and miR-301a null irradiated with lethal dose were transplanted with 10 million WT BM cells to generate chimeric mice and colitis was induced. Immuno-histochemical staining show enhancement of E-cadherin expression in miR-301a−/− chimeras compared to WT chimeras. *In vivo* permeability analysis shows the lesser decrease of intestinal permeability and significantly less production of IL1b, IL6, and TNFα in IECs of miR-301a−/− chimeras in comparison with WT chimeras after DSS treatment. Further, in *in vitro* cell culture model, overexpression of miR-301a in HCT116 inhibits transepithelial electrical resistance, promotes FITC permeability, and ethyl-hydroxyethyl cellulose translocation indicating the confirmatory role of miR-301a in the modulation of IB dysfunction. In conclusion, these data show that expression of miR-301a promotes mucosal permeability and multiple downstream effectors of miR-301a in the regulation of IBD and colitis-induced colon cancer (18). In conclusion, these data demonstrate the critical roles of miR-301a in the disruption of the mucosal immunology of the gastrointestinal tract.

miR-301a knockout mice are viable with no apparent developmental disorder, and thus it will be of paramount importance to see the long-term effect of miR-301a ablation in inflammation-associated disorder. Given its critical role in cancer progression, it will be interesting to see if miR-301a plays any role in growth and renewal of gastrointestinal cancer stem cell. Furthermore, whether perturbation of normal gut microbiota affects miR-301a expression in a mouse model is not studied. Moreover, whether miR-301a expression can be used as a biomarker for IBD, IBD-associated cancer, and marker for IBD therapy in a large cohort of patient needs to be evaluated. Importantly, whether miR-301a has any implication in non-IBD-associated colon cancer is not evaluated. In comparison to other ncRNA, miR-301a seem to be a better target because it regulates both STAT3 and NF-κB signaling which induce pattern recognition receptors, such as toll-like receptor and NOD-like receptor, antigen receptors of lymphocytes, and several cytokines (19). One of the attractive strategies will be to deliver viral-mediated antisense miR-301a into the intestinal epithelial cell (IEC) and CD4+ T-cells which are critical in regulating early events, such as barrier injury, NF-κB activation, and TNFα production. In spite of well established function of miR-301a in inflammation-associated malignancies, one of the considerable challenges in miR-301a-based therapy would be to design the efficient delivery tool with cell-specific targeting, avoid off-target effects, and prevent potential toxicities in the clinical trial (20). In summary, the future studies will uncover the detailed understanding of miR-301a on the regulation of mucosal immunity and hold the promise for treating patients with IBD and autoimmune disorders.

## Author Contributions

PG and SR wrote the first draft. SB conceived, planned, and finalized the manuscript.

## Conflict of Interest Statement

The authors declare that the research was conducted in the absence of any commercial or financial relationships that could be construed as a potential conflict of interest.
